# The neuroblast and angioblast chemotaxic factor SDF-1 (CXCL12) expression is briefly up regulated by reactive astrocytes in brain following neonatal hypoxic-ischemic injury

**DOI:** 10.1186/1471-2202-6-63

**Published:** 2005-10-31

**Authors:** Jason T Miller, John H Bartley, Hereward JC Wimborne, Aisha L Walker, David C Hess, William D Hill, James E Carroll

**Affiliations:** 1 Department of Neurology, Medical College of Georgia, Augusta, GA 30912, USA; 2 Department of Pediatrics, Medical College of Georgia, Augusta, GA 30912, USA; 3 Veteran's Affairs Medical Center, 1 Freedom Way, Augusta, GA 30904, USA; 4 Department of Cellular Biology & Anatomy, Medical College of Georgia, Augusta, GA 30912, USA

## Abstract

**Background:**

Stromal cell-derived factor 1 (SDF-1 or CXCL12) is chemotaxic for CXCR4 expressing bone marrow-derived cells. It functions in brain embryonic development and in response to ischemic injury in helping guide neuroblast migration and vasculogenesis. In experimental adult stroke models SDF-1 is expressed perivascularly in the injured region up to 30 days after the injury, suggesting it could be a therapeutic target for tissue repair strategies. We hypothesized that SDF-1 would be expressed in similar temporal and spatial patterns following hypoxic-ischemic (HI) injury in neonatal brain.

**Results:**

Twenty-five 7-day-old C57BL/J mice underwent HI injury. SDF-1 expression was up regulated up to 7 days after the injury but not at the later time points. The chief sites of SDF-1 up regulation were astrocytes, their foot processes along blood vessels and endothelial cells.

**Conclusion:**

The localization of SDF-1 along blood vessels in the HI injury zone suggests that these perivascular areas are where chemotaxic signaling for cellular recruitment originates and that reactive astrocytes are major mediators of this process. The associated endothelium is likely to be the site for vascular attachment and diapedesis of CXCR4 receptor expressing cells to enter the injured tissue. Here we show that, relative to adults, neonates have a significantly smaller window of opportunity for SDF-1 based vascular chemotaxic recruitment of bone marrow-derived cells. Therefore, without modification, following neonatal HI injury there is only a narrow period of time for endogenous SDF-1 mediated chemotaxis and recruitment of reparative cells, including exogenously administered stem/progenitor cells.

## Background

Hypoxic-ischemic (HI) injury to the brain is a significant cause of neurological morbidity in infants. The resultant severe and longstanding disability to infants has provided impetus for exploring aggressive therapies that give promise of injury reversal. Transplantation of stem cells has emerged as a possible modality to help improve function in damaged tissues [1].

In organ-specific injury models, bone marrow cells have been shown to accumulate at the site of injury and to differentiate into tissue specific cells [2-5]. Chen and colleagues [6] reported that intravenously administered marrow stromal cells accumulate in the site of ischemic brain injury, with a small percentage expressing neuronal markers. Additionally, Hess et al. [7,8] have demonstrated that, following temporary ischemia, endogenous murine bone marrow cells give rise to perivascular/mural cells, microglial cells, and a limited number of cells positive for the neuronal marker, NeuN. However, it is unclear if the NeuN positive cells develop into mature neurons.

Among the milieu of chemicals expressed in the injured microenvironment, the chemotactic chemokine, stromal cell derived factor-1 (SDF-1 or CXCL12), has emerged as a major attractant of reparative cells. SDF-1 and its receptor, CXCR4, play a critical role in both engraftment of hematopoietic stem cells into bone marrow and the mobilization of stem/progenitor cells from bone marrow niches into the peripheral circulation where these cells are then available for delivery to sites of peripheral injury [9-11]. In addition to functioning in the hematopoietic system, CXCR4 positive cells respond to SDF-1 signals in non-hematopoietic injured organs [12]. That exogenous bone marrow-derived cells home to and migrate within the site of injury suggests that the brain's response to injury produces signals that attract these cells [7,8,13]. Functionally, SDF-1 is a powerful chemoattractant for CXCR4-expressing bone marrow derived cells, including putative bone marrow stem/progenitor cells such as CD34+ cells both in vitro and in vivo [14].

In addition to targeting hematopoietic cells, the SDF-1/CXCR4 system is also known to play a critical role in embryonic brain development and adult injury repair including neurogenesis, neuroblast migration, and neuronal organization as well as endothelial progenitor cell recruitment, endothelial cell migration and vasculogenesis [15-25].

Therefore, it is important to determine the spatial and temporal expression of SDF-1 relative to injury; SDF-1 expression patterns may establish not only the location and duration of the window of opportunity for endogenous injury-mediated chemotaxis of reparative cells, but also the timeframe for therapeutic transplantation and recruitment of various stem cell populations. Recently, we showed in an adult mouse model of stroke that SDF-1 is expressed perivascularly in the injured region up to 30 days after middle cerebral artery occlusion [13], suggesting that there may be a long post-injury window for homing of reparative cells. Anticipating that SDF-1 might be a critical factor in attracting transplanted cells to the injured area of the neonatal brain, we hypothesized that SDF-1 would be expressed in neonatal hypoxic-ischemic injury in a temporal and spatial pattern similar to that seen in adult models of ischemic brain injury.

## Results

### Spatial expression pattern of SDF-1 in uninjured brain

**Figure 1:**
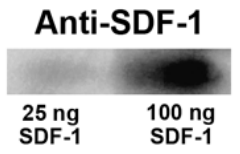
**SDF-1 Western blot**. Recombinant murine SDF-1α (25 or 100 ng per lane) was run on a one-D gel, transferred to PVDF membrane and probed with the SDF-1 antibody used in the immunohistochemical assays. The western blot demonstrates that the SDF-1 antibody recognizes murine SDF-1α.

**Figure 2:**
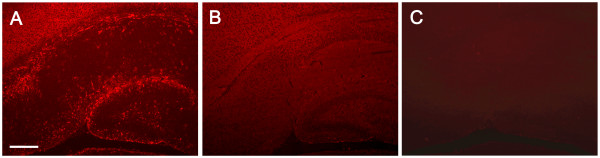
**SDF-1 Antibody Specificity**. The polyclonal SDF-1 antibody was tested for non-SDF-1 antigenic interactions and non-specific tissue binding. Panel A demonstrates the SDF-1 antibody's recognition of SDF-1 antigen on mouse hippocampus. Panel B demonstrates that preabsorption of the SDF-1 antibody with recombinant murine SDF-1α completely blocks specific binding of the antibody to the tissue with only very low (diffuse background) non-specific binding. Panel C shows that leaving the primary (SDF-1) antibody incubation step out of the staining protocol does not result in non-specific binding of the secondary antibody or the detection reagents. Scale bar equals 200 μm.

**Figure 3:**
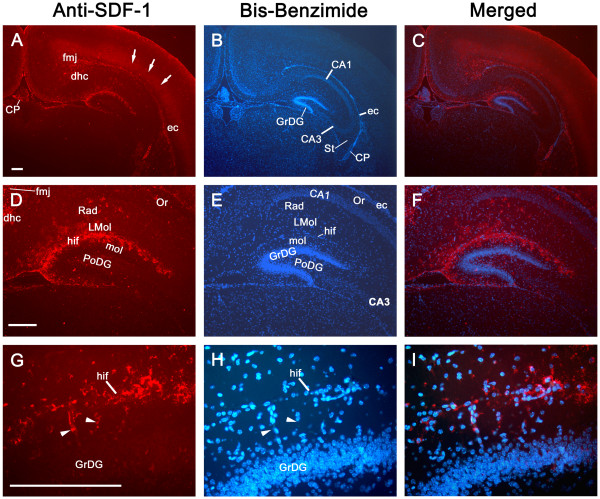
**Constitutive SDF-1 protein expression at P8**. The left column shows SDF-1 antibody staining of P8 mouse brain (Cy3 – red). The middle column shows the corresponding nuclear labelling (bis-benzimide – blue) of the same sections. The right hand column shows the merged images. Panels A-C show the distribution of constitutive SDF-1 expression at low power. The arrows denote diffuse labelling over the neocortex. Panels C-E demonstrate the hippocampal SDF-1 expression pattern. Panels G-I show a high magnification of the hippocampal fissure down through the upper blade of the dentate gyrus granular cell layer. The arrowheads point out SDF-1 positive blood vessels. Abbreviations: ca – cornus ammons (CA1, 2, 3), cp – choroid plexus, dhc – dorsal hippocampal commissure, ec – external capsule, fi – fimbria hippocampus, fmj – forceps major corpus callosum, GrDG – granular layer dentate gyrus, hif – hippocampal fissure, LMol – lacunosum moleculare layer hippocampus, Mol – molecular layer dentate gyrus, Or – oriens layer, PoDG – polymorph layer dentate gyrus, Rad – stratum radiatum. All Scale bars equal 200 μm.

**Figure 4:**
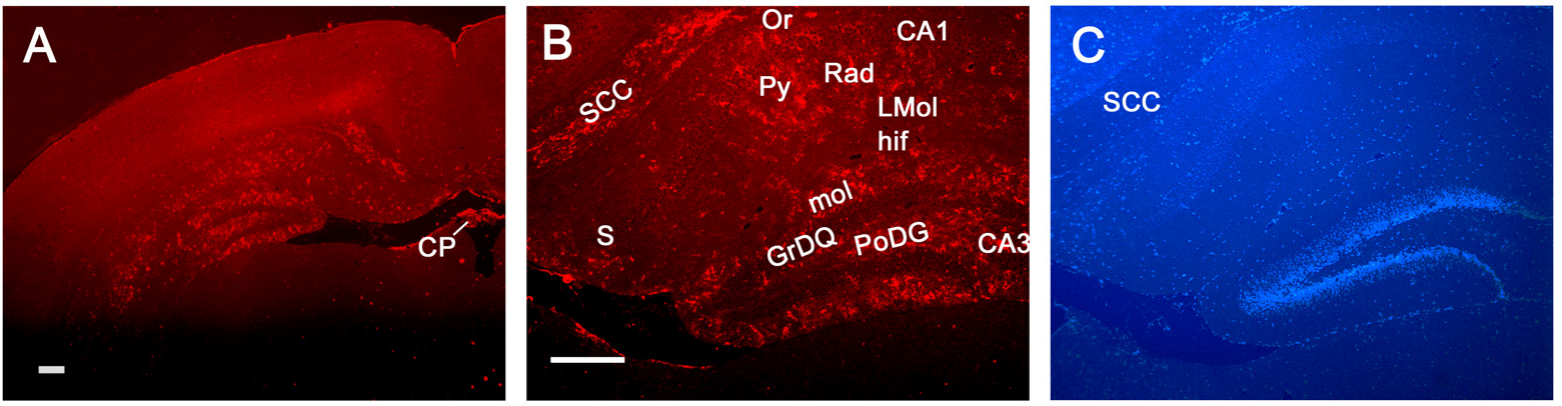
**Constitutive SDF-1 protein expression at P14**. Panel A shows the pattern of SDF-1 expression (Cy3 – red) at P14 (Scale bar equals 200 um). Panel B demonstrates the hippocampal expression of SDF-1 (Cy3 – red) (Scale bar equals 200 μm). Panel C shows bis-benzimide nuclear labelling (blue) from the same field as Panel B. Abbreviations: ca – cornus ammons (CA1, 2, 3), cp – choroid plexus, GrDG – granular layer dentate gyrus, hif – hippocampal fissure, LMol – lacunosum moleculare layer hippocampus, Mol – molecular layer dentate gyrus, Or – oriens layer, PoDG – polymorph layer dentate gyrus, Py – pyramidal cell layer, Rad – stratum radiatum. S – subiculum, scc – splenium corpus callosum.

**Figure 5:**
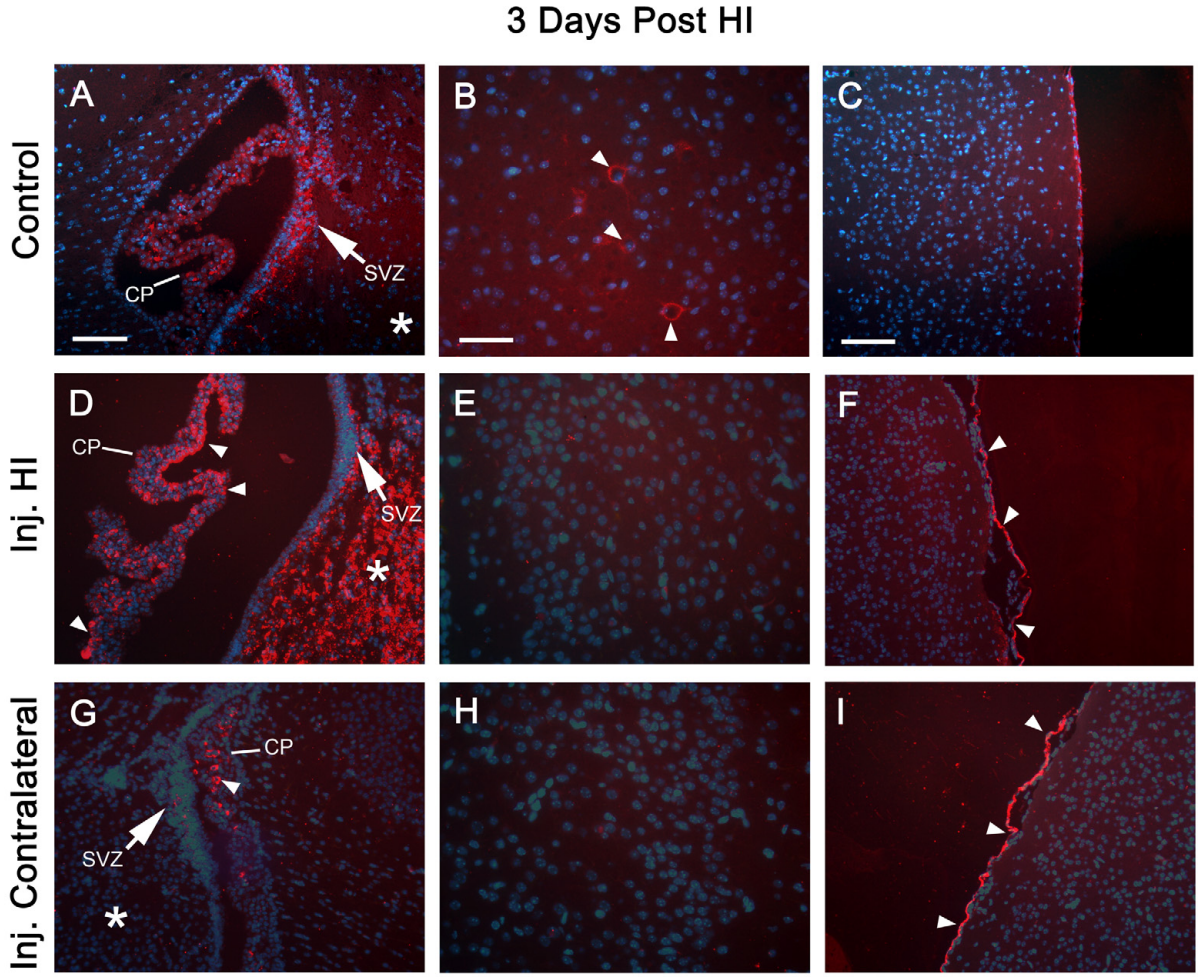
**Constitutive and Injury Induced Expression of SDF-1**. All panels are from P10 murine neonates. Panels A-C are from a non-Hypoxia-Ischemia (HI) control animal; D-I are from an animal that underwent HI injury three days previously at P7. Panels D-F are from the HI injured hemisphere and G-I from the corresponding contralateral (non-ischemic) hemisphere. Panels A, D, and G are of the lateral ventricle including the CP, SVZ (arrow) and adjacent striatum (*); arrowheads indicate SDF-1 expression (Cy3 – red) in the CP, scale bar equals 100 μm. Panels B, E, & H are of the cingulate cortex, arrowheads indicate SDF-1 expression in neurons, scale bar equals 50 μm. Panels C, F, and I show cortex and meninges, arrowheads indicate SDF-1 expression in mesothelial cells of the pia, scale bar equals 100 microns. Cell nuclei were identified by bis-benzimide counterstaining (blue). Abbreviations: choroid plexus (CP) and subventricular zone (SVZ).

The SDF-1 antibody used for immunohistochemical localization of SDF-1 recognizes full length recombinant murine SDF-1 (Figure 1) and preabsorption with SDF-1 blocks its recognition of antigen on tissue sections (Figure 2). In uninjured (control) neonatal mice (P8 through P17) endogenous protein expression of SDF-1 in brain is limited and changes over time (Figures 3, 4, 5). At P8 (Figure 3) the most prominent SDF-1 expression is within the hippocampus associated with the hippocampal fissure and the neighbouring molecular layer of the dentate gyrus, the hippocampal lacunosum molecular layer, the dentate gyrus polymorph layer, the stratum radiatum and the oriens layer. Strong expression is also associated with cells in the choroid plexus, the neurogenic subventricular zone, and large cortical neurons in the cingulate and neocortex. Additionally, large white matter tracks such as the corpus callosum and external capsule, as well as cells of the meninges, also demonstrate SDF-1 expression. Within the white matter tracks and the hippocampal fissure numerous blood vessels are associated with SDF-1 labelling (Figure 3). However, unexpectedly, the granular cell layer of the dentate gyrus, CA and layer V neurocortical neurons do not show strong immunohistochemical staining for SDF-1 (Figure 3). There is some shift in SDF-1 protein expression with time. By P14 the hippocampal expression changes slightly (Figure 4). Expression associated with the hippocampal fissure is reduced while there is increased expression in hippocampal areas associated with dendritic arborization and axonal fields, including the dentate gyrus polymorph layer, the molecular layer of the dentate gyrus, the hippocampal lacunosum molecular layer and the stratum radiatum (Figure 4).

### Changes in spatial expression following HI injury

**Figure 6:**
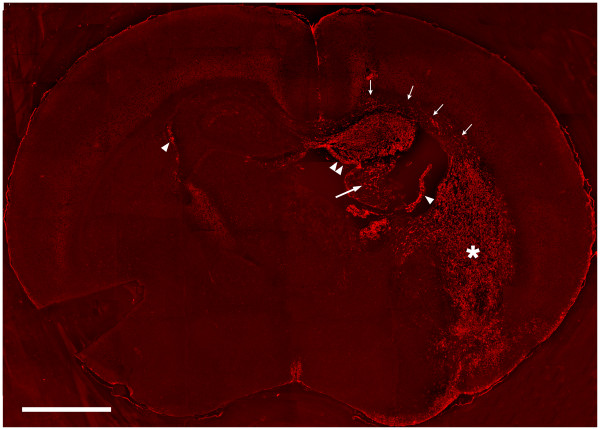
**SDF-1 Expression 3 days Post Hypoxia-Ischemia**. A coronal section from a P10 mouse that underwent hypoxia-ischemia (with right side ischemia) three days earlier, on postnatal day 7, was probed with a Cy3 (red) antibody to demonstrate SDF-1 expression. Single arrowheads indicate choroid plexus, double arrowheads the hippocampus, the large arrow indicates the fimbria of the hippocampus, small arrows the corpus callosum and external capsule, the asterisk (*) denotes the striatum. All five animals at this time point showed equivalent levels of SDF-1.

**Figure 7:**
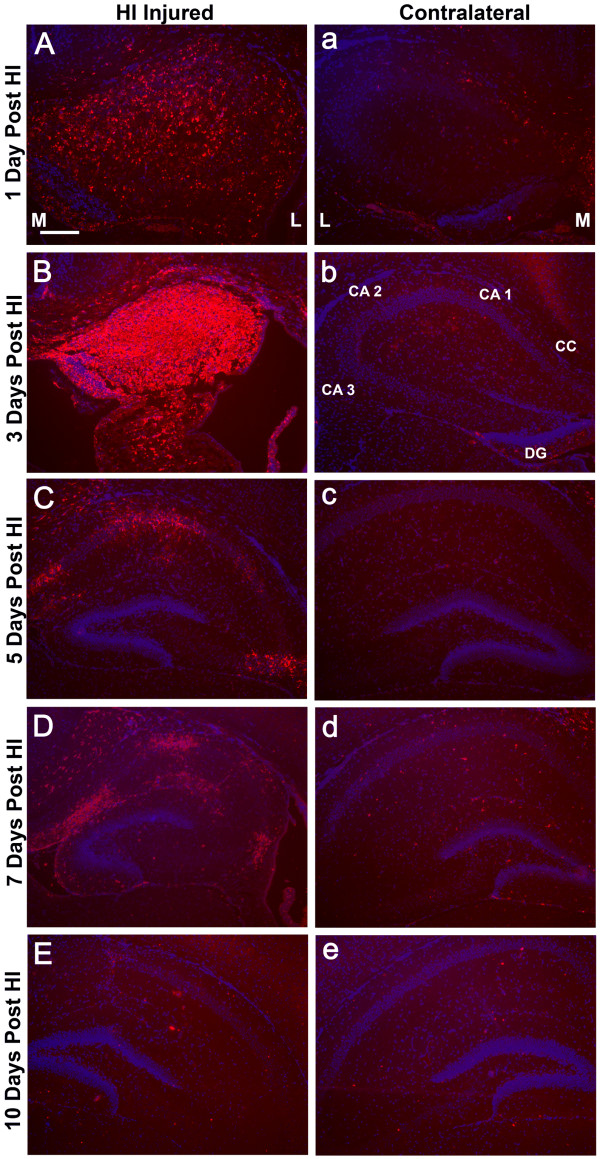
**Temporal Expression of SDF-1 in Hippocampus Post-HI**. Shows examples of hippocampal expression of SDF-1 at different time points post hypoxia-ischemia (HI) demonstrated by immunohistochemistry (Cy3 label – red), this includes 1 (P8), 3 (P10), 5 (P12), 7 (P14) and 10 (P17) days following HI. Panels A-E are from the ischemic hemisphere and a-e are from the contralateral hemisphere. M and L denote the medial and lateral aspects of the hippocampi respectively. Hippocampal regions are indicated: cornu ammonis (CA 1, 2 & 3), corpus callosum (CC), and dentate gyrus (DG). Cell nuclei were identified by bis-benzimide counterstaining (blue). Scale bar equals 200 μm.

Following HI injury the endogenous constitutive expression in the large neurons of the cingulate and neocortex is lost. However, at the same time SDF-1 expression is dramatically up regulated in not only areas directly affected by HI, but also in the choroid plexus and by mesothelial cells of the pial meninges (Figures 5 and 6). The expression of SDF-1 in areas of HI injury principally includes the hippocampus and striatum (figures 6 and 7). Additionally, there is increased and extensive expression by cells within the HI side hemisphere white matter, particularly in the corpus callosum, external capsule and hippocampal fimbria (Figure 6). Interestingly, the choroid plexus and meninges expression of SDF-1 increases bilaterally with HI injury, although the degree of expression in the contralateral choroid plexus appears to be less robust than the HI side choroid plexus (Figures 5 and 6). This suggests a generalized injury response that extends beyond the area of ischemia but potentially within the area affected by hypoxia.

### Temporal expression pattern of SDF-1 following HI injury

The first day after HI injury (P8) patchy SDF-1 expression was demonstrated around blood vessels and within the parenchyma in the hippocampus and striatum within the ischemic hemisphere. By 3 days after HI injury (P10) there was robust, uniform hippocampal expression of SDF-1 in the CA1, CA2, CA3 and dentate regions as well as within the striatum and to a limited degree in the cingulate cortex (Figures 5, 6, 7). By day 5 after the injury (P12) SDF-1 continued to be expressed but was significantly reduced in the hippocampus and striatum. However, its expression was still increased in the choroid plexus (bilaterally) and was associated with blood vessels in the white matter of the corpus callosum and external capsule as well as pial mesothelial cells (bilaterally). By day 7 (P14), SDF-1 expression continued to wane, and by day 10 post-HI injury (P17) and beyond, expression was reduced below observable levels in the hippocampus, striatum and cortical areas. The only expression of SDF-1 at these late times was found along white matter tracts of the corpus callosum and the alveus and in some sites where normal constitutive expression is observed, specifically the subventricular zone, choroid plexus and a subset of large cingulate cortex and neocortical neurons. However, expression appeared to be depressed in hippocampal areas that normally expressed SDF-1.

### Cellular expression of SDF-1

**Figure 8:**
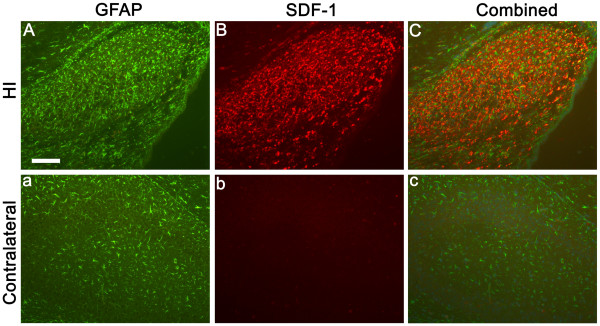
**SDF-1 and GFAP Co-localization**. Panels A-C are from a P10 hippocampus, three days post-HI, from the ischemic hemisphere, a-c are from the corresponding contralateral hippocampus. Panels A & a demonstrate the astrocyte marker GFAP (FITC – green). Panels B & b show SDF-1 expression (Cy3 – red). Panels C & c indicate the overlap between GFAP and SDF-1 labeling. Scale bar equals 100 μm.

**Figure 9:**
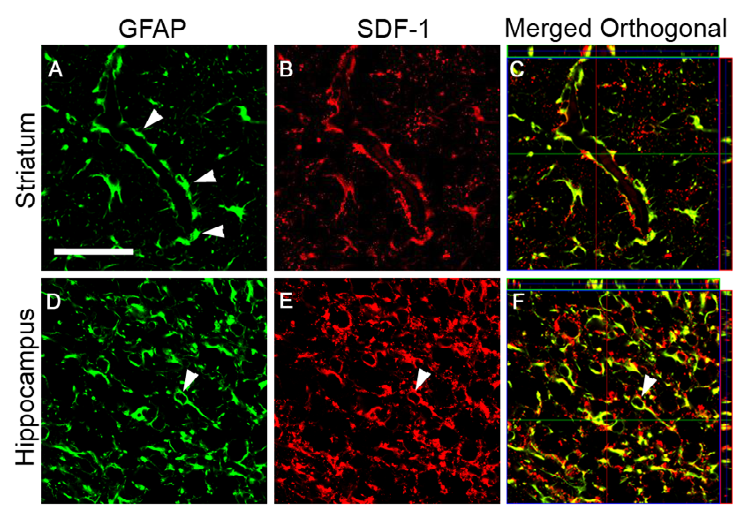
**Astrocyte Expression of SDF-1**. GFAP and SDF-1 dual labeling is demonstrated by confocal microscopy in P10 mice, three days post-HI. Panels A-C, from the striatum, show perivascular (arrowheads) astrocyte (GFAP – green) expression of SDF-1 (red). Panels D-F, from the hippocampus, show (arrowhead) similar co-labeling of GFAP (green) astrocyte cell bodies and processes for SDF-1 (red). Panels C and F show GFAP and SDF-1 co-labeling in merged orthogonal views of confocal z stacks. GFAP staining is shown in green and SDF-1 staining in Red. Panels A, B, D, and E are single z plane field images captured with a 63× objective. Scale bar equals 50 μm.

**Figure 10:**
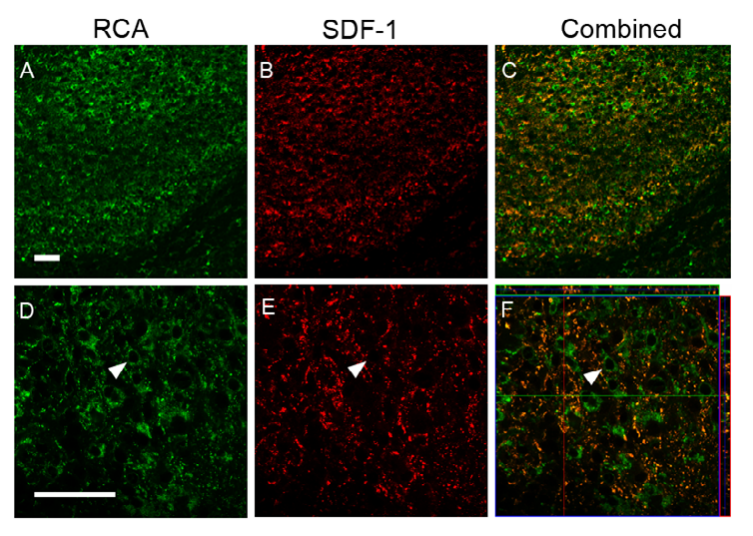
**SDF-1 and RCA-1 Co-localization**. Figure shows RCA and SDF-1 dual labeling in hippocampus from P10 mice three days post-HI; RCA-1 labeling in hippocampus (A & D), SDF-1 expression in hippocampus (B & E), merged RCA-1 and SDF-1 labeling (C & F). Panels A-C represent 20× objective fields. Panels D-E are single z plane fields and panel F is a merged orthogonal view of a confocal z stack. The arrowhead points out an example of an SDF-1 negative RCA-1 positive cell. Scale bar equals 50 μm.

For all time points demonstrating injury responsive SDF-1 expression, cellular expression was in large part localized to GFAP+ reactive astrocytes (Figures 8 and 9). Astrocyte associated expression of SDF-1 is frequently found around small blood vessels, suggesting that this perivascular expression is related to astrocytic foot processes (Figures 8 and 9). The lectin RCA-1 labels cell surface carbohydrate moieties associated with both microglial and endothelial cells. The punctate RCA+/SDF-1+ labelling resembles cellular processes, however, the cell type demonstrating this co-labeling is uncertain, morphologically it does not resemble GFAP+/SDF-1+ astrocytic cell processes or endothelial cell profiles (Figure 10). Some of the SDF-1+/RCA-1+ profiles do appear to be ramified microglial cells. However, while a subset of microglia in the HI injury area co-label with SDF-1 the majority do not (data not shown). Further, SDF-1 did not co-label with markers for other process bearing cell types including oligodendrocytes (data not shown). Also, as described earlier, choroid plexus and pial epithelial cells demonstrate SDF-1 expression (Figures 3, 5 and 6).

## Discussion

The regulation of SDF-1 may be critical to the incorporation of a variety of new cells into brain. In contrast to the prolonged up regulation of SDF-1 following stroke in adult mice [13], its up regulation in neonatal mice after HI injury was brief. This may reflect a difference between a mature, relatively stable, adult brain structure and a more plastic, still developing, neonatal CNS where there may be a more active regulation of SDF-1 expression. The timing of post-HI injury SDF-1 expression suggests that SDF-1 mediated cell homing may be most effective from 3 to 5 days after HI injury in neonatal animals, peaking around 3 days. During this time SDF-1 expression is high around vascular elements, which may provide a portal of access for circulating reparative cells to the areas of ischemic injury.

Another possible explanation for the temporal differences of SDF-1 expression between injured adult and neonatal brains may be related to the type of injury. HI injury produces a more diffuse and graded tissue injury, while middle cerebral artery occlusion is more focal and discrete.

### Constitutive SDF-1 expression

Interestingly, the pattern of peak expression for SDF-1 we show at postnatal day 10, 3 days after HI injury (Figure 6), is virtually identical to that recently published by Felszeghy et al for I^125 ^SDF-1α binding to CXCR4 in a rat HI model, 2 days after HI injury, at postnatal day 9 [26]. In Felszeghy et al's study the localization of CXCR4 receptors suggests the distribution of SDF-1 expression. Protein expression observed in our study, with SDF-1 antibody probes, is consistent with mRNA expression patterns assayed by in situ hybridization in previous reports with some exceptions [26-28]. The SDF-1 mRNA expression patterns Tham et al and Stumm et al [22,23] show at days P7 and P9 in rats match protein expression patterns we detected here at P8 and P10 in mice (Figures 3, 5 and 6). This includes SDF-1 expression in the meninges, the hippocampal fissure, choroid plexus and the proliferative subventricular zone. SDF-1 mRNA expression in both in situ hybridization studies [22,23] was also associated, to a limited degree, with blood vessels, we saw the same constitutive pattern for SDF-1 immunostaining particularly with vessels associated with the hippocampal fissure and with white matter tracks (Figure 3). In the in situ hybridization studies [22,23] there is a decline in hippocampal fissure SDF-1 mRNA expression between the early time points (P7 & P9) and later time points (P14 and P12 – P21). A similar decrease in hippocampal fissure SDF-1 immunostaining was observed in our study between P8 and P14 (Figures 3 and 4). Of interest, in the *in situ *hybridization studies [22,23], there are consistent increases in SDF-1 mRNA in the dentate gyrus granular layers, in CA3 (and CA4) and in layer V neocortical neurons between the early time points (P7 & 9) and later time points (P14 and P12 – P21). However, we did not detect significant SDF-1 immunolabelling over the cell bodies of the granular cells at equivalent time points in the mice (P8 – 14). In contrast we did detect immunolabelling in the molecular and polymorph layers, regions of granular cell dendritic arborizations and axon fields [27]. This labelling increased between P8 and P14 (Figures 3 and 4). Similarly we did not detect strong SDF-1 immunoreactivity over CA 3, 2 or 1 neuronal somas, but we found immunoreactivity that increased between P8 and P14 in the hippocampal lacunosum molecular layer, the stratum radiatum and the oriens layer (Figures 3 and 4), regions that contain the dendritic and axonal fields for the CA region [27]. Additionally, the layer V neocortical neurons are reported to show strong mRNA labelling [22,23], but we only detected weak diffuse antibody labelling in the area corresponding to layers V and VI in the neocortex (Figure 3).

The discrepancy between the reported neuronal somal labelling for mRNA [22-24] and the neuropil immunolabelling for SDF-1 protein we observed may be due to a lack of sensitivity of the antibody, although we can clearly detect SDF-1 expressing structures that are reported to express lower levels of SDF-1 mRNA than dentate gyrus granular cells, CA and layer V neocortical neurons [22,23]. There may also be differences in SDF-1 transcription and translation rates as well as message and protein turnover in different cell populations. Alternatively, this may reflect that SDF-1 mRNA is synthesized in, and restricted to, the neuronal somas while in some neurons the protein product is mainly exported to cellular processes with little remaining in the somas. For example, while the reported dentate gyrus granular cell mRNA expression [22-24] is more intense than any other location, including the hippocampal fissure, we show that this is at best poorly labelled immunohistochemically (Figures 3 and 4). In contrast we demonstrate that regions that reflect the synaptic fields (dendritic and axonal) of the granular cells (molecular and polymorph layers) have strong immunoreactive expression for SDF-1 (Figures 3 and 4) while these same regions show little mRNA expression for SDF-1 [22-24]. Further, structures lacking projecting processes, which could distance mRNA form protein localization, like the hippocampal fissure, choroid plexus, and meninges, are reported to be strongly labelled for SDF-1 mRNA by in situ hybridization [22-24] and we demonstrate also by immunohistochemistry for the SDF-1 protein. There is precedent for this type of pattern in neonatal hippocampus. Czarnecki et al have recently shown that synaptopodin mRNA was restricted to dentate granular layer, and CA3, cell somas while immunostaining for synaptopodin protein labeled dendritic layers but not the somas [28].

### Injury induced SDF-1 expression

Recently Ceradini et al. demonstrated that SDF-1 gene expression is regulated by the hypoxia responsive transcription factor HIF-1 [21]. The level of SDF-1 expression is directly related to the degree of hypoxia such that hypoxic gradients within tissues correlate with gradients of SDF-1 gene expression [21]. We have previously shown in an adult middle cerebral artery occlusion model of stroke that SDF-1 expression follows a gradient pattern consistent with the hypoxia gradient in the penumbra [13]. In our neonatal HI model the hippocampus did not appear to show a gradient of SDF-1 labeling during peak expression, rather the labeling was uniform and delimited by anatomical boundaries. In contrast the striatal expression did appear to show a gradient of labeling intensity (Figure 6). Specific cell types may be more sensitive to hypoxic changes in inducing SDF-1 expression as illustrated by changes in constitutive expression of SDF-1 as well as induced expression in cells that did not show significant constitutive expression. For example, subsets of neocortical neurons responded by decreasing SDF-1 expression in both the hypoxic and HI affected hemispheres while choroid plexus and pial labeling increased on both sides and astrocytes dramatically expressed SDF-1 only in the HI hemisphere. This suggests that HIF-1 expression may be differentially induced or that other transcription factors may be involved as well.

### Potential role of SDF-1 in injury repair

SDF-1 and its receptor CXCR4 are emerging as a common axis for directing the migration of stem cells associated with injury repair in many systems. Ji et al recently demonstrated that SDF-1 mediates the migration of mesenchymal stem cells to the site of injury [29]. Neural progenitor cells and neuroblasts express CXCR4 and are attracted by SDF-1α developmentally and following brain ischemia [25,30-32]. Recently SDF-1 has been shown to promote neovascularization in somatic tissues and to recruit bone marrow-derived (BMD) cells to areas of ischemic injury. In mouse models of peripheral tissue ischemia, SDF-1 treated animals had increased capillary density and blood flow compared to controls and recruited injected endothelial precursor cells (EPC) to integrate into blood vessels demonstrating vasculogenesis [16,20,21]. Importantly this SDF-1 induced neovascularization has been demonstrated to improve perfusion and function in peripheral tissue ischemic injury models [16,21,33]. Work from our laboratory shows SDF-1 is up regulated in the penumbral, or peri-infarct, zone within hours of acute ischemia in adult mice. This elevated expression persists in the peri-infarct zone for at least 30 days post-ischemia. Accompanying this we observe bone marrow-derived cell infiltration, pericyte and microglial engraftment and neovascularization [13]. Given the apparent function of SDF-1 to serve as an attractant for bone marrow-derived cells, including cells associated with vascular repair and vasculogenesis, as well as for other stem/progenitor cells, including neural stem cells, this brief time span of SDF-1 expression, peaking at 3 days with only limited expression through 7 days after the injury in neonatal animals, may be critical for the acquisition of new brain or reparative cells.

The cell type expressing SDF-1 and its localization is highly related to its function. Previously, we found that the principal localization of SDF-1 expression following injury was astrocytic, often strongly associated with small blood vessels [13]. This was the first demonstration that activated, or reactive astrocytes, express SDF-1 *in vivo *following ischemia injury [13]. The current study confirms and extends our original finding and demonstrates that SDF-1 expression is massively up regulated in reactive astrocytes in neonatal HI injury, but for a shorter period of time relative to adult middle cerebral artery occlusion ischemia. Recently Imitola [25] also reported SDF-1 expression in reactive astrocytes in an adult HI model. Earlier *in vitro *studies showed that cultured astrocytes up regulate SDF-1 expression following exposure to various cytotoxic or inflammation-inducing agents [34,35]. Additionally, we have recently shown that there is only minimal proliferation and maturation of astrocytes following HI, suggesting that the astrocytic expression of SDF-1 following HI originates from existing astrocytes [36].

The influence of SDF-1 upon ischemic recovery in neonates appears to be restricted to a few days under circumstances of "normal" endogenous repair. Such a brief up regulation of SDF-1 in neonatal animals may disadvantage their recovery relative to adults. However, it may still may provide a defined time frame in which therapeutically transplanted cells, or autologous stem cells mobilized from the bone marrow, may be able to home, engraft and differentiate into needed cell types. This may also provide an opportunity to target engineered cells that express specific factors to the site of injury. Using the SDF-1/CXCR4 axis as a vectoral targeting system may also permit local application of cell expressed therapeutic factors in higher doses than systemic delivery would safely allow. In adults it may be possible to use the natural up regulation of the SDF-1 for up to 30 days after the injury, while in neonates such a procedure would have to be carried out much sooner. If transplantation protocols are carried out in humans, it may be necessary to find ways of either up regulating the SDF-1 or engineering the expression of the chemokine to desired temporal and spatial locals.

## Conclusion

SDF-1 up regulation following neonatal HI injury persists only a few days in comparison to the much longer up regulation in adult models of stroke. The up regulation in neonatal HI injury is quite prominent in astrocytes abutting small blood vessels. This process may be critical in attracting CXCR4 expressing cells to the brain. Awareness of these events could be helpful in planning stem cell transplantation therapy delivered via the vascular route.

## Methods

### Animals

This study was performed in accordance with the guidelines provided by the Laboratory Animal Studies Committee of the Veterans Administration Medical Center (Augusta, GA) and the Medical College of Georgia. The mice were housed in individual cages under standard conditions. Offspring were reared with their dams until the time of surgery, and then until weaning at three weeks of life.

### Surgical procedure and treatment

Twenty-five C57 BL/6 mouse pups underwent permanent ligation of the left common carotid artery at post-natal day seven (P7) [37]. A midline cervical incision was made to expose the left common carotid artery, and a single suture ligated the artery. The incision was closed with interrupted 6-0 silk sutures. During the procedure, the mice were anesthetized with 2% isoflurane. The pups were then placed with the dams for two hours prior to placement in an 8% oxygen chamber partially immersed in a water bath at 37° for 75 minutes.

### Animal sacrifice and tissue processing

The pups were sacrificed at P8, P10, P12, P14, P17, P21, or P35 (respectively, 1, 3, 5, 7, 10, 14 or 28 days after the injury). At least three animals were sacrificed at each time point. At the time of sacrifice, the animals were anesthetized with 70 mg/kg of ketamine and 15 mg/kg xylazine. Tissue was fixed by transcardiac perfusion with 0.9% saline, followed by 4% paraformaldehyde in PBS. Brains were removed and post-fixed in 4% paraformaldehyde for 4 hours, cut into 3–5 mm sections, and continued in 4% paraformaldehyde for an additional 20 hours. Tissue was then dehydrated in an ethanol series, cleared in xylene, and infiltrated and embedded in PolyFin Embedding and Infiltration Wax (TBS Biomedical Sciences, Durham, NC). Paraffin embedded tissue was sectioned 5 μ thick and mounted on Superfrost Plus slides (Fisher Scientific).

### Immunohistochemistry

Section processing, antigen retrieval and immunostaining methods have been previously described [13,36] Briefly, slide mounted sections were deparaffinized through 3 xylene changes followed by rehydration in an alcohol dilution series. Sections were permeablized in 0.1% Triton-X 100 in PBS for ten minutes, followed by 3 washes in 1× PBS. Antigen retrieval was performed using a microwave defixation method. Slides were microwaved at a slow boil for 10 minutes in citrate buffer (0.01 M sodium citrate pH 6.5) then incubated at rest for twenty minutes, then washed in 3 changes of 1× PBS and blocked using 2% normal calf serum in PBS for 20 minutes at room temperature (RT).

Sections were labeled with antibodies to SDF-1 (Santa Cruz cat # sc-6193, goat, recognizes both SDF-1α & β splice variants). SDF-1 signal was detected by utilizing either a biotin/avidin (Vector Elite ABC kit) or peroxidase anti-peroxidase (PAP; Sternberger) amplification system and visualized with Cy3 conjugated antibody (Jackson ImmunoResearch Laboratories). In some instances dual labeling for SDF-1 and a second antigen was utilized to identify the phenotype of SDF-1 expressing cells. For co-labeling SDF-1 and the astrocyte marker glial fibrillary acidic protein (GFAP) a rabbit anti-GFAP antibody (DAKO cat# Z0334) was used, for oligodendricytes anti-CNPase (Sigma cat# C5922, mouse) and the biotinylated lectin *Ricinus Communis Agglutinin I *(RCA 1, Vector cat# B-1085, detects unblocked N-Acetyl galactosamine and galatose) for the visualization of endothelial cells and microglial cells [13,36]. All antibodies (and RCA-1) were allowed to incubate at room temperature for one hour and washed four times (1× PBS 5 minutes each). All primary antibodies were detected using indirect staining with a secondary, fluorescent antibody. Cy3 anti-Goat (Jackson # 705-165-147), diluted 1:400, was used to visualize SDF-1 in all cases. FITC anti-rabbit (Jackson # 711-095-152), diluted 1:100, was used to visualize GFAP. Strepavidin FITC (Jackson #016-010-084), diluted 1:100, was used to visualize biotinylated RCA. FITC anti-mouse (Jackson #715-095-151), diluted 1:100, was used to visualize CNPase. Slides were rinsed in 4 changes of PBS for 5 minutes each. Negative controls consisted of preabsorbing 660 ng/ml, (4.3 pM/ml) the SDF-1 antibody (Santa Cruz cat # sc-6193, goat) with 1.5 ng/ml (43 pM/ml) of recombinant murine SDF-1α (Peprotech # 250-20A) at 37°C for 1 hour prior to use or leaving off the primary antibody. Cell nuclei were counterstained with bis-benzimide (Sigma, 10 mg/ml stock diluted 1:12,000 in phosphate buffered saline) for 8 minutes then given a final wash prior to coverslipping with Vectashield mounting media (Vector Labs #H-1000).

### Western blotting

Recombinant murine SDF-1α (25 & 100 ng) was run on a Novex NuPage 10% Bis-Tris Gel (Invitrogen NP0302BOX) with MES buffer at 75 volts then transferred to a 0.2 um PVDF membrane (Millipore Immobilon PSQ #ISEQ00010). The blot was blocked with 4% cold water fish gelatin (Sigma #G-7765) in PBS at room temperature for 1 hour. The SDF-1 antibody (Santa Cruz cat # sc-6193, goat) was incubated shaking (1:100, 10 ug/ml) overnight at 4°C. The blot was rinsed, then washed 2 times for 10 minutes each in PBS with 0.5% Tween-20 (Fisher #BP337-500). The anti-SDF-1 antibody was detected with an anti-goat secondary antibody conjugated with HRP (Jackson ImmunoResearch #705-035-003, 1:50,000) for 1 hour at room temperature. The blot was washed again (3 times for 10 minutes each in PBS with 0.5% Tween-20 and 1 time with PBS only), developed with ECL detection reagent (Amersham # RPN2109D1) and exposed to X-Ray film (Fujifilm Super RX #100NIF).

### Confocal imaging

Confocal imaging was used to confirm dual labeling with a Zeiss 510 laser scanning confocal microscope (Zeiss LTM software) as previously described [13,36]. The objective used was Zeiss 63× C-Apochromat 1.2NA. To excite the FITC fluorochrome (green), a 488-nm laser line generated by an argon laser was used, and for the Cy3 fluorochrome (red), a 543-nm laser from a HeNe laser was used. Filter sets used were a bandpass 500- to 600-filter ("green" channel) and a long-pass 585- to 650-nm filter ("red" channel). We identified cells that were labeled for SDF-1 and either GFAP or RCA with 1-μm step Z series.

## Authors' contributions

JM, HW, and AW performed the tissue cutting, staining and section assessments. JM also assisted in writing the manuscript. DH participated in the conception of the project and in evaluation of the results. WDH participated in the conception of the project, directed the immunohistochemical studies, assisted in evaluation of the results and in drafting the manuscript. JC developed and directed the overall project, evaluated the results, and drafted the manuscript.
